# Factors associated with risk perception among school and nursery school teachers concerning potential health effects of exposure to electromagnetic fields

**DOI:** 10.3389/fpubh.2026.1944031

**Published:** 2026-09-18

**Authors:** Katharina Lüthy, Claudia Riesmeyer, Jessica Kühn, Estelle Blättermann, Felix Forster, Katja Radon, Tobias Weinmann

**Affiliations:** 1 Institute and Clinic for Occupational, Social and Environmental Medicine, LMU Medizin, LMU University Hospital, Ludwig-Maximilians-Universität München, Munich, Germany; 2 Department of Media and Communication, Ludwig-Maximilians-Universität München, Munich, Germany

**Keywords:** electromagnetic fields, environmental exposures, nursery school teachers, risk perception, school teachers

## Abstract

**Background:**

There is considerable concern in the public about potential health risks from exposure to electromagnetic fields (EMF) below legal limits. School and nursery school teachers are important multipliers of health-related information, also regarding environmental exposures like EMF. Factors associated with the risk perception towards EMF in these professional groups have not yet been investigated.

**Methods:**

This 2024 cross-sectional study surveyed school teachers (*N* = 1,053) and nursery school teachers (*N* = 329) across Germany to examine factors associated with participants’ EMF risk perception. We applied logistic regression modelling to examine the relationship between EMF risk perception (high vs. low) and the variables technology acceptance, media health literacy, conspiracy belief, trust in various organizations and institutions as well as general environmental worry.

**Results:**

About one-third of the participants believed that some individuals develop adverse health effects from EMF exposure below legal limits. Conspiracy belief was positively associated with high EMF risk perception (school teachers: OR: 2.87, 95% CI: 2.36–3.43; nursery school teachers: OR: 2.88, 95% CI: 2.00–4.00). Technology acceptance (school teachers: OR: 0.47, 95% CI: 0.39–0.55; nursery school teachers: OR: 0.69, 95% CI: 0.51–0.89), media health literacy (only in school teachers (OR: 0.71, 95% CI: 0.61–0.82)), and trust in authorities like WHO were inversely associated with EMF risk perception.

**Conclusion:**

Our findings underline that individuals’ appraisal of scientific evidence, in this case concerning EMF exposure, is related not only to individual skills like media health literacy, but also to personal attitudes like the tendency towards conspiracy beliefs or trust in organizations.

## 1 Introduction

Due to the widespread use of technologies like Wi-Fi and devices like smartphones or electronical household appliances, non-ionizing electromagnetic fields (EMF) ranging from extremely low frequency to radiofrequency fields are omnipresent in modern society. Exposure to EMF can have biological effects on the human body like the stimulation of nerve cells or an increase in tissue temperature (1–3). These thermal effects have no demonstrable adverse impact on health when legal exposure limits are adhered to (4–6). The exposure of the general population to EMF is usually far below these legal limits (2, 7, 8).

Nonetheless, there are discussions about possible non-thermal effects on human health below legal exposure limits. For example, long-term exposure to extremely low-frequency magnetic fields (ELF-MF) in the general Swiss population was associated with dementia mortality; however, only limited causal conclusions could be drawn from this study (9). Other research observed an association between ELF-MF and childhood leukemia, also without providing evidence for a causal link (10). Moreover, recent studies found no association between radiofrequency EMF exposure and risks of glioma, non-Hodgkin lymphoma, or thyroid cancer (11–13). Among Swedish and Finnish participants, a link between long cell phone calls and insomnia appeared driven by usage-related psychological factors, not EMF exposure itself (14). A recent systematic review found no causal evidence that exposure to EMF is associated with the non-specific endpoints of tinnitus, migraine, headaches, and sleep disturbances, though uncertainty remains due to the small number of studies, a potential risk of bias in some studies, as well as inconsistencies and imprecision (15).

At the same time, surveys report considerable levels of concern in the general population about potential health risks in relation to EMF exposure. In a study conducted in 2015 across seven European countries, participants were asked to rate how dangerous they consider EMF exposure situations such as cell phone calls, being in the immediate vicinity of Wi-Fi devices and cell phone base stations on a Likert-scale from 1 = “not dangerous” to 5 = “very dangerous” (16). The average ratings were around 3 (16). In a study conducted in 2022 among the general population in Germany, about a fifth of the participants stated to be very worried about radiation arising from smartphones, cell phone base stations, and high-voltage power lines, while about half of the respondents said that they felt unable to protect themselves from these types of radiation (17).

Scientific publications are often difficult to understand for non-experts (18), which may contribute to discrepancies between scientific evidence and public concern (19). Effective risk communication and knowledge transfer among scientists, experts, science communicators, and (health) authorities are therefore essential to reduce uncertainty and prevent misconceptions (20). To adequately inform the public about potential EMF-related health risks, it is important to understand both the nature of these concerns and the factors contributing to their development (21).

School and nursery school teachers are important multipliers who disseminate information, including health-related information, to children, parents, and the wider public (22, 23). As key agents of socialization, they also convey knowledge and skills related to environmental influences such as EMF. Consequently, they represent an important interface between scientific knowledge and the public regarding potential health effects of EMF exposure.

Despite this role, factors associated with EMF risk perception have not yet been investigated among school and nursery school teachers. EMF receive only limited attention in German school curricula and are rarely addressed in teachers’ education, except in physics teachers. As primary school and nursery school teachers generally encounter the topic of potential health effects in relation to EMF neither during training nor in professional practice, their engagement with the topic is likely to be limited. Accordingly, both the extent of EMF risk perception in these professional groups and the factors shaping remain largely unknown. Some understanding of variables associated with EMF risk perception stems from studies among physicians (24, 25). In this group, technology acceptance (TA), media health literacy (MHL), conspiracy belief, trust in different organizations/institutions/initiatives, and environmental worry are discussed as being possibly related EMF risk perception (25, 26).

TA is the process of evaluating a new technology’s utility and ease of use to decide on its adoption (27). Accordingly, there are some studies reporting an association between the acceptance of new technologies and their perceived usefulness. For example, studies on 5G suggested that its acceptance increased when users perceived it as useful, easy to use, and compatible with their digital skills (28). Another study observed an inverse association between individual openness to technological progress and risk perception regarding 5G (29). Against this background, TA was included in the presented study to examine whether higher TA is associated with a lower perception of EMF risk.

The same applies to MHL which is defined as the literacy to identify and critically analyze health-related content from various media, to recognize its influence on health behavior, and to react through action (30). There are indications that information about EMF in the media sometimes contradicts the current state of scientific research and that false reports and fake theories are often circulating in the media—for example about alleged harmful health effects of EMF in relation to 5G (31). Negative or incorrect information can influence an individual’s opinion and, for example, lead to health anxieties and mistrust (32). We hypothesized that higher MHL is associated with lower EMF risk perception, as false information in the media might be better recognized as incorrect, while scientifically accurate information might be more easily found and evaluated.

Conspiracy theories explain events or social conditions through the hidden actions of powerful groups. Behind these secret machinations lies the suspected intention of achieving malicious goals—such as gaining political power—by breaking rules and manipulating information (33, 34). There are also numerous conspiracy theories circulating about technologies that generate EMF, such as the theory that EMF exposure from 5G devices can both cause COVID-19 and increase its severity, and that mobile communications can weaken the human immune system (35, 36). Furthermore, there is some evidence from previous studies that conspiracy belief may be associated with EMF risk perception (25, 37).

Trust in regulatory authorities, companies, and the scientific community has been reported as being associated with the acceptance of EMF-generating technologies such as mobile communications (26, 38–40). Conversely, distrust may lead to heightened risk perception and demands for stricter regulation. In this sense, trust is the willingness to rely on those responsible for decisions and actions related to technology management or other areas of public health and safety (20, 39, 40).

Environmental worry is defined as the emotional component of general environmental attitudes. While environmental attitudes encompass a person’s entire body of knowledge and views regarding the environment, environmental concern specifically describes the worry or unease felt in the face of environmental problems (41). Previous studies investigated whether risk perception regarding EMF stems from specific engagement with EMF exposure or whether it is an expression of general environmental risk perception (25, 26).

This study therefore sought to investigate factors associated with EMF risk perception among school teachers and nursery school teachers.

Specifically, we examined the following hypotheses:

H1: A higher level of TA is associated with lower EMF risk perception.

H2: A higher level of MHL is associated with lower EMF risk perception.

H3: A higher level of conspiracy belief is associated with higher EMF risk perception.

H4: Trust in organizations/initiatives/institutions is associated with EMF risk perception (direction of association depends on type of organization/initiative/institution).

H5: EMF risk perception is positively associated with general environmental worry.

## 2 Methods

We conducted a cross-sectional online survey across Germany collecting data from June to December 2024. The survey was approved by the Ethics Committee at the Medical Faculty of LMU Munich (project number 24–0204). All participants gave informed consent to take part in the study. In an earlier analysis from the same survey, we provided information on the prevalence of EMF risk perception and the sociodemographic characteristics of the study population (42).

### 2.1 Study population

The study population consisted of school teachers and nursery school teachers from various educational institutions that completed a standardized questionnaire. Using convenience sampling, study invitations were distributed via social media, newspapers advertising, and by contacting relevant federal/state associations requesting them to spread information about the study. Furthermore, 2,400 postal invitations were sent to quasi-randomized school facilities and day nurseries in every state, also asking them to spread information about the study among their staff. As an incentive, participants received a shopping voucher worth 25 EUR for completing the questionnaire.

### 2.2 Measures

The questionnaire was based on previous, comparable studies (26, 43) and was administered via LimeSurvey. To determine the comprehensibility and feasibility of the questionnaire as well as the average completion time, a pre-test was carried out before the actual survey. This pre-test confirmed the planned response time of approximately 15 min, as well as the clarity of the questions. Both the original German version and a translation into English are provided in Supplementary file 1.

#### 2.2.1 Outcome

The primary outcome was participants’ EMF risk perception, defined as an individual’s subjective risk assessment (44). Applying the same item as earlier studies investigating EMF-related risk perception among physicians (26, 43), participants were asked to indicate their agreement to the following statement “There are individuals who develop adverse health effects from electromagnetic fields below legal limits” (hereafter: EMF health effects below legal limits) with answer options on a 5-point-Likert-scale ranging from 0 = “I do not agree” to 4 = “I agree.” For statistical analysis, answers above the central option were categorized as high risk perception and answers from 0–2 as low risk perception. The classification of the central answer option as low risk perception was done in line with the approach of previous studies and to not overestimate the degree of agreement (26, 43). Dichotomizing this item is in line with the analytical approach by the studies among physicians (26, 42). Besides, we considered this approach as potentially useful to correct for differences in response patterns between participants assuming that some people may be less likely than others to select an extreme category, even though their level of agreement does not actually differ (“tendency toward the middle”).

To account for the analytical loss of information that comes along with dichotomization, we performed a sensitivity analysis that used the original 5-point item as outcome variable.

#### 2.2.2 Independent variables

TA was assessed with 12 statements regarding 5G (45), e.g., “The use of 5G services will have more benefits than disadvantages”. The degree of agreement was also measured with a 5-point-Likert-scale. Afterwards, a sum score was calculated from all 12 items, which could therefore have a value between 0 and 48. A higher score corresponded to a higher TA.

A similar process was used for the variable MHL. Here, participants were asked for their personal assessment of eight statements, which could be rated from “very difficult” to “very easy” using a 5-point Likert-scale (0–4). The statements referred to personal literacy regarding information searches and information evaluations on the internet, e. g., “How easy/difficult is it for you to visit different websites to check if they contain the same information on the topic?” (46). The sum score could range from 0 to 32, with higher values indicating higher MHL.

Conspiracy belief was measured using the Flexible Inventory of Conspiracy Suspicions (47, 48). Participants’ agreement to five statements was asked, e. g., “The truth is that the association between health issues and EMF is hidden from the public”. Again, answer options were provided on a 5-point-Likert-scale ranging from 0 = “I do not agree” to 4 = “I agree”. The sum score ranged from 0 to 20 with higher values indicating higher tendency towards conspiracy belief.

All three of these scales had excellent internal consistency with a Cronbach’s alpha in the entire study population of 0.92 for the TA scale, 0.86 for the MHL scale, and 0.90 for the scale measuring conspiracy belief.

Trust in different organizations (World Health Organization (WHO), Federal Office for Radiation Protection, Federation of German Consumer Organizations) and institutions (Federal government, politicians, political parties, courts, large companies, police, armed forces, media, science, most other people) was also taken into account as being potentially associated with EMF risk perception. The measurement was done using a Likert scale from 0 to 4, with participants selecting one category from “not trustworthy” to “very trustworthy” and “I do not know this organization” (for organizations) or from “no trust” to “very high trust” (for institutions). This operationalization of institutional trust follows established distinctions in communication research, differentiating between media, social, political, and societal trust (49). The term “media” refers broadly to trust in various forms of media objects, including media brands, different types of media or mainstream journalism, without differentiating between different offerings. “Science” captures perceptions of both individual scientists and the scientific community. Finally, “most other people” reflects perceived public opinion and general societal trust. These institutional actors also communicate messages in diverse ways and at different levels, both individually and as part of organizations, thereby addressing a range of audiences. In addition to trust in organizations and institutions, trust in “EMF critical” initiatives (Citizen initiatives, pleas from EMF skeptical physician initiatives, Institute for Applied Ecology) was also assessed. No sum score was formed from these variables because the individual organizations and institutions were too different in terms of content. Instead, the respective mean value of each item was calculated with higher mean values indicating higher trust in the respective organization/institution. The answer option “I do not know this organization” was not included in the calculation of the mean score.

The question “Do you worry about your personal health because of…” was used to measure general environmental worry. With a 5-point-Likert-scale, the participants were asked about their concern regarding air pollution, traffic noise, drinking water pollution, impacts of climate change, resistance to antibiotics, and pesticides (26, 43). Participants who answered for at least three of these items above the central answer option were considered as having high environmental worry. Otherwise, participants were considered as having low/no environmental worry.

#### 2.2.3 Additional variables

We also collected information on the following socio-demographic variables: gender, age, Federal state, type of municipality (i.e., number of inhabitants) in which participants’ institution is located, and sponsorship of their institution (public or private). Additionally, school teachers were asked about the type of school they work at.

### 2.3 Statistical analysis

Descriptive statistics with respect to sociodemographic characteristics are shown both for the study population (all study participants) and the source population (population from which the participants came), the latter based on aggregated data provided by the Federal Office for Statistics. Associations between the independent variables and the outcome EMF risk perception (high vs. low) were examined using separate logistic regression models, reporting odds ratios (OR) and 95% confidence intervals (CI). Continuous variables (TA, MHL, and conspiracy belief) were standardized ((sum score—mean value)/standard deviation), environmental worry was included as a dichotomous variable, and trust variables were analyzed using their original Likert-scale values (0–4) with participants giving the answer option “I do not know this organization” being excluded from the models on trust variables. All models were adjusted for age, gender, sponsorship of institution, type of municipality and Federal state of the working place. Separate models were run for school teachers vs. nursery school teachers because of marked difference between the two groups with respect to sociodemographic characteristics, particularly socio-economic status.

As a sensitivity analysis, we repeated all models as ordinal logistic regression using the original 5-point Likert item for EMF risk perception (answer options ranging from 0 = “I do not agree” to 4 = “I agree) as endpoint. As another sensitivity analysis, we conducted a binary logistic regression model with mutual adjustment for TA, MHL and conspiracy belief to account for potential interrelatedness between these variables.

All models were performed as complete-case analyses.

Data management and statistical analyses were conducted in R, including the packages “rstanarm” (50), “brms” for ordinal logistic regression (51, 52) and the Bayesian analysis software Stan (53). Estimates were summarized by median (point estimate) and 95%-highest posterior density intervals, which is the narrowest interval that contains 95% of posterior probability (interval estimate). For each model, 2,000 samples per chain (with 1,000 of them being warm-up samples) were drawn from four chains, leading to 4,000 usable samples. We used the weakly informative default priors of the rstanarm and brms packages. Diagnostic criteria (effective sample size, $\hat{R}$ , tree depth, energy (E-BFMI), and divergent transitions) were inspected and indicated no problems.

## 3 Results

### 3.1 Descriptives

1,053 school teachers and 329 nursery school teachers took part in the online survey (Table 1).

Most participants were female and aged between 30 and 49 years More details on the sociodemographic characteristics of the study population and the entire source population are provided elsewhere (42).

**Table 1:** Distribution of age and gender in the study population.

Variable	School teachers	Nursery school teachers
Total	1,053 (75.2%)	329 (23.5%)
Gender		
Female	666 (63.2%)	256 (77.8%)
Male	380 (36.1%)	70 (21.3%)
Diverse	5 (0.5%)	3 (0.9%)
*Missing (n)*	*2*	*0*
Age group		
< 30 years	105 (10.0%)	78 (23.7%)
30–39 years	333 (31.6%)	103 (31.3%)
40–49 years	304 (28.9%)	81 (24.6%)
50–59 years	230 (21.8%)	54 (16.4%)
≥ 60 years	81 (7.7%)	13 (4.0%)
*Missing (n)*	*0*	*0*

Further details on sociodemographic characteristics of the study population and the entire source population can be found elsewhere (42).

Concerning the outcome EMF-related risk perception, 31.0% agreed with the statement regarding EMF health effects below legal limits, with no differences between school teachers (31.2%) and nursery school teachers (31.6%).

Furthermore, TA and MHL had mean values of 25.3 (SD: 11.9) and 23.2 (SD: 5.7), respectively (Table 2). Trust in WHO, Federal Office for Radiation Protection, and Federation of German Consumer Organizations was high in both professional groups (mean: 2.9, 3.2, and 3.0 respectively). Citizens’ initiatives received the lower trust ratings across both professions (mean: 2.0; SD: 1.4). Science received the highest trust ratings within the institutions, averaging 3.4 (SD: 0.8) among school teachers and 2.7 (SD: 1.1) among nursery school teachers.

**Table 2:** Descriptive statistics for the independent variables.

Variable	Missing	Total	School teachers	Nursery school teachers
	*n*	mean (SD)	mean (SD)	mean (SD)
Technology acceptance	124	25.3 (11.9)	25.6 (11.9)	24.4 (12.2)
Media health literacy	126	23.2 (5.7)	23.9 (5.5)	20.7 (5.7)
Conspiracy belief	169	6.0 (5.4)	5.0 (5.0)	9.3 (5.3)
Trust in organizations				
WHO	167	2.9 (1.1)	2.9 (1.0)	2.4 (1.2)
Federal Office for Radiation Protection	166	3.2 (1.1)	3.3 (0.9)	2.3 (1.3)
Federation of German Consumer Organizations	167	3.0 (0.9)	3.1 (0.9)	2.5 (1.0)
Trust in initiatives				
Institute for applied ecology	169	2.6 (1.0)	2.7 (1.0)	2.3 (1.0)
Physicians’ appeals	166	2.9 (1.1)	3.0 (1.1)	2.4 (1.1)
Citizens’ initiatives	166	2.0 (1.4)	2.1 (1.5)	1.9 (1.1)
Trust in institutions				
Federal government	169	2.2 (1.1)	2.4 (1.0)	1.5 (1.1)
Politicians	170	1.9 (1.1)	2.1 (1.1)	1.3 (1.0)
Political parties	170	1.9 (1.1)	2.1 (1.1)	1.4 (1.0)
Courts	169	2.9 (1.0)	3.1 (0.9)	2.4 (1.1)
Large companies	171	1.4 (1.1)	1.5 (1.1)	1.2 (1.0)
Police	169	2.8 (0.9)	2.9 (0.9)	2.6 (1.0)
Armed forces	169	2.5 (1.0)	2.6 (1.0)	2.4 (1.0)
Media	168	1.9 (1.1)	2.0 (1.1)	1.5 (1.1)
Science	169	3.2 (0.9)	3.4 (0.8)	2.7 (1.1)
Most other people	170	2.2 (0.9)	2.3 (0.9)	2.0 (0.9)
Environmental worry: high	179	n (%) 707 (57.9)	n (%) 517 (55.8)	n (%) 178 (63.6)

Scale limits: Technology acceptance: 0–48; Media Health Literacy: 0–32; Conspiracy belief: 0–20; Trust in organizations: 0–4; Trust in initiatives: 0–4; Trust in institutions: 0–4.

While nursery school teachers had a mean value of 9.3 (SD: 5.3) on the conspiracy belief scale—suggesting that nursery school teachers on average selected the middle answer category on the 5-point Likert-scale for each of the five statements—the mean score among school teachers was 5.0 (SD: 5.0), which corresponds to an average choice of the second lowest answer option at this item.

Over half of all participants (57.9%) reported being concerned about various environmental factors affecting their personal health. Nursery school teachers were slightly more likely to be concerned (63.6%) than school teachers (55.8%). The greatest concerns among participants were related to the effects of climate change with 41.5% selecting the highest answer option.

### 3.2 Logistic regression

A one-standard-deviation increase in TA was inversely associated with EMF risk perception among school teachers (OR: 0.47, 95% CI: 0.39–0.55) and nursery school teachers (OR: 0.69, 95% CI: 0.51–0.89) (Table 3). The same—though to a lesser extent—was observed with MHL which was also inversely associated with EMF risk perception (school teachers: OR: 0.71, 95% CI: 0.61–0.82; nursery school teachers: OR: 0.93, 95% CI: 0.70–1.19). Moreover, greater trust in the Federal Office for Radiation Protection was linked to lower EMF risk perception (school teachers: 0.65, 95% CI: 0.56–0.76; nursery school teachers: 0.86, 95% CI: 0.71–1.02), as was trust in WHO (school teachers: OR: 0.71, 95% CI: 0.61–0.81; nursery school teachers: OR: 0.93, 95% CI: 0.75–1.11). Higher trust in the Federal government, politicians, political parties and science was also inversely associated with EMF risk perception. Furthermore, a one-standard-deviation increase in conspiracy belief scores was associated with an almost threefold higher odds ratio of high EMF risk perception in both groups (school teachers: OR: 2.87, 95% CI: 2.36–3.43; nursery school teachers: OR: 2.88, 95% CI: 2.00–4.00). High environmental worry was associated with nearly twice the odds of high EMF risk perception among school teachers (OR: 1.90, 95% CI: 1.35–2.53), but not among nursery school teachers (OR: 0.98, 95% CI: 0.49–1.65).

**Table 3:** Adjusted binary logistic regression models for the association between the independent variables and EMF risk perception (binary outcome).

Variable	School teachers			Nursery school teachers
	*n*	OR (95% CI)*	*n*	OR (95% CI)*
Technology acceptance	959	0.47 (0.39–0.55)	293	0.69 (0.51–0.89)
Media health literacy	957	0.71 (0.61–0.82)	293	0.93 (0.70–1.19)
Conspiracy belief	925	2.87 (2.36–3.43)	282	2.88 (2.00–4.00)
Environmental worry (worried vs. not worried)	920	1.90 (1.35–2.53)	278	0.98 (0.49–1.65)
Trust in organizations				
World Health Organization	926	0.71 (0.61–0.81)	283	0.93 (0.75–1.11)
Federal Office for Radiation Protection	926	0.65 (0.56–0.76)	284	0.86 (0.71–1.02)
Federation of German Consumer Organizations	926	0.77 (0.65–0.89)	283	1.17 (0.93–1.48)
Trust in initiatives				
Institute for Applied Ecology	923	1.00 (0.89–1.11)	284	1.00 (0.83–1.19)
Physicians’ appeals	926	0.98 (0.88–1.10)	284	1.13 (0.93–1.38)
Citizens’ initiatives	926	1.12 (1.02–1.23)	284	1.09 (0.93–1.27)
Trust in institutions				
Federal government	925	0.67 (0.58–0.78)	282	0.62 (0.46–0.79)
Politicians	923	0.69 (0.59–0.79)	283	0.62 (0.45–0.81)
Political parties	924	0.66 (0.56–0.76)	282	0.58 (0.42–0.76)
Courts	924	0.79 (0.67–0.93)	283	0.87 (0.67–1.08)
Large companies	924	0.68 (0.57–0.78)	281	1.03 (0.77–1.34)
Police	924	0.96 (0.80–1.13)	283	0.91 (0.69–1.16)
Armed forces	924	0.89 (0.75–1.03)	283	1.10 (0.81–1.40)
Media	925	0.71 (0.61–0.82)	283	0.61 (0.45–0.80)
Science	925	0.53 (0.43–0.64)	282	0.62 (0.48–0.80)
Most other people	924	0.77 (0.65–0.91)	282	1.26 (0.93–1.65)

*Adjusted for age, gender, sponsorship of institution, type of municipality and federal state.

CI: 95%-highest posterior density intervals.

n: model-specific complete-case sample size.

In the ordinal logistic regression using the original 5-point item for EMF risk perception as endpoint, technology acceptance was inversely associated with EMF risk perception in school teachers (OR: 0.46, 95% CI: 0.40–0.53) and nursery school teachers (0.70, 95% CI, 0.55–0.86). The estimates for the association of MHL with EMF risk perception was OR: 0.70 (95% CI, 0.62–0.80) in school teachers and OR: 0.87 (95% CI, 0.69–1.06) in nursery school teachers. A standard-deviation increase in the conspiracy belief scores was associated with EMF risk perception in both groups (school teachers: OR: 3.27; 95% CI: 2.77–3.80); nursery school teachers: OR: 2.37 (95% CI, 1.80–2.98). Also for the variables concerning trust in the different institutions and organizations, the OR and 95% CI from the ordinal logistic regression were very similar to the estimates provided by the binary logistic regression (Table 4).

**Table 4:** Adjusted ordinary logistic regression models for the association between the independent variables and EMF risk perception (ordinal outcome).

Variable	School teachers		Nursery school teachers	
	*n*	OR (95% CI)*	*n*	OR (95% CI)*
Technology acceptance	959	0.46 (0.40–0.53)	293	0.70 (0.55–0.86)
Media health literacy	957	0.70 (0.62–0.80)	293	0.87 (0.68–1.06)
Conspiracy belief	925	3.27 (2.77–3.80)	282	2.37 (1.80–2.98)
Environmental worry (worried vs. not worried)	920	2.15 (1.62–2.72)	278	0.92 (0.53–1.40)
Trust in organizations				
World Health Organization	926	0.67 (0.59–0.76)	283	0.90 (0.75–1.05)
Federal Office for Radiation Protection	926	0.63 (0.55–0.72)	284	0.81 (0.69–0.93)
Federation of German Consumer Organizations	926	0.71 (0.62–0.81)	283	1.02 (0.82–1.23)
Trust in initiatives				
Institute for applied ecology	923	1.02 (0.92–1.10)	284	1.00 (0.86–1.14)
Physicians’ appeals	926	0.97 (0.89–1.06)	284	1.02 (0.87–1.18)
Citizens’ initiatives	926	1.14 (1.05–1.23)	284	1.13 (1.00–1.28)
Trust in institutions				
Federal government	925	0.64 (0.56–0.72)	282	0.63 (0.50–0.77)
Politicians	923	0.64 (0.57–0.72)	283	0.61 (0.47–0.76)
Political parties	924	0.65 (0.57–0.73)	282	0.57 (0.45–0.72)
Courts	924	0.73 (0.64–0.84)	283	0.73 (0.58–0.89)
Large companies	924	0.71 (0.62–0.80)	281	1.06 (0.82–1.30)
Police	924	0.90 (0.78–1.03)	283	0.77 (0.60–0.93)
Armed forces	924	0.83 (0.73–0.94)	283	0.91 (0.72–1.13)
Media	925	0.62 (0.55–0.71)	283	0.66 (0.52–0.81)
Science	925	0.52 (0.43–0.60)	282	0.55 (0.43–0.68)
Most other people	924	0.71 (0.61–0.82)	282	1.30 (0.97–1.63)

*Adjusted for age, gender, sponsorship of institution, type of municipality and federal state. CI: 95%-highest posterior density intervals.

n: model-specific complete-case sample size.

In the sensitivity with mutual adjustment for TA, MHL and conspiracy belief, TA remained associated with EMF risk perception in both groups (school teachers: OR: 0.58, 95% CI: 0.48–0.71); nursery (school teachers: OR: 0.72, 95% CI: 0.52–0.95). The same applies to conspiracy belief school teachers: (OR: 2.51, 95% CI: 2.02–3.02); nursery school teachers: (OR: 2.92, 95% CI: 1.93–4.14). For MHL, meanwhile, there was no evidence for an association with EMF risk perception after adjustment for TA and MHL, neither in school teachers (OR: 1.00, 95% CI: 0.83–1.18) neither in nursery school teachers (OR: 1.00, 95% CI: 0.70–1.13) (Table 5).

**Table 5:** Binary logistic regression models with mutual adjustment for technology acceptance, media health literacy and conspiracy belief.

Variable	School teachers		Nursery school teachers	
	*n*	OR (95% CI)	*n*	OR (95% CI)
Technology acceptance ^a^	925	0.58 (0.48–0.71)	282	0.72 (0.52–0.95)
Media health literacy ^b^	925	1.00 (0.83–1.18)	282	1.00 (0.70–1.33)
Conspiracy belief ^c^	925	2.51 (2.02–3.02)	282	2.92 (1.93–4.14)

a Adjusted for media health literacy, conspiracy belief, age, gender, sponsorship of institution, type of municipality and federal state.

b Adjusted for technology acceptance, conspiracy belief, age, gender, sponsorship of institution, type of municipality and federal state.

c Adjusted for technology acceptance, media health literacy, age, gender, sponsorship of institution, type of municipality and federal state.

CI: 95%-highest posterior density intervals.

## 4 Discussion

### 4.1 Main findings

This study aimed to identify factors associated with EMF risk perception among school and nursery school teachers. In binary as well as ordinal logistic regression models, we observed strong evidence for a positive association between conspiracy belief and EMF risk perception, while TA and MHL were inversely associated with EMF risk perception. Greater trust in official bodies and institutions like the Federal government, politicians, science, or radiation protection authorities was also inversely associated with EMF risk perception. General environmental worry was associated with EMF risk perception only among school teachers.

The most striking finding was that among both school and nursery teachers, conspiracy belief was associated with EMF risk perception, even after adjustment for technology acceptance and media health literacy. Evidence for an association between individuals’ tendency towards conspiracy beliefs and EMF risk perception has also been observed among physicians (25). Various studies suggest that people tend to turn to conspiracy theories when important psychological needs—such as coping with anxiety, uncertainty, and threat—are not met (54, 55). Belief in conspiracy theories can influence the acceptance of medical measures—for example, the willingness to get vaccinated, adherence to treatment plans, or even the willingness of affected individuals to seek help (56, 57). Furthermore, belief in a conspiracy theory can also have an impact on a believer’s social behavioral patterns. For instance, they distance themselves from the community and from those who do not believe in the conspiracy (54, 58). Social isolation, in turn, could constitute a risk factor for mental illness and may lead to an increased incidence of negative health outcomes such as anxiety or depression (59, 60). Furthermore, belief in conspiracy theories could not only endanger the health of the general population—for instance, through the refusal of vaccinations—but could also lead to increased treatment costs, heightened morbidity, and, above all, mortality, by delaying the initiation of necessary medical treatment. The impact of belief in conspiracy theories on public health can therefore be classified as substantial (61).

It should also be noted that the conspiracy belief score among school and nursery school teachers measured in this study, with mean scores of 5.0 and 9.3 respectively, was higher than the mean score of 4.2 among physicians in the study among physicians mentioned above (25). Given the wording of the questions used to assess conspiracy belief, the mean score among nursery school teachers can be considered relatively high. Studies suggest that belief in conspiracy increases, among other things, as education levels decrease (62, 63), which may at least partly explain the higher scores among nursery school compared to physicians and school teachers. A more direct explanation for the higher conspiracy belief score among nursery school teachers compared to school teachers is that they more frequently used alternative media as source of information than school teachers as observed in an earlier analysis from the present dataset (42).

Most participants (58%) reported high general environmental worry, which was greater than their concern regarding EMF. This may indicate that other environmental issues are perceived as more concerning or that the benefits of EMF-related technologies outweigh their risks. Similarly, a survey of the German general population found that concerns were highest towards antibiotics and pesticides in food, air pollution, and multi-resistant bacteria, while most participants perceived high-voltage power lines as beneficial for themselves and for society (64). Nevertheless, school teachers with high environmental worry reported greater EMF risk perception than those with lower environmental worry. One explanation may be that EMF are viewed as part of broader environmental concerns. In addition, the ongoing digitization of schools, including widespread Wi-Fi access and increasing use of interactive whiteboards, may heighten awareness of environmental, EMF-related, and health issues, particularly given school teachers’ responsibility for children. The absence of this association among nursery school teachers may be interpreted as indication that they may perceive EMF separately from broader environmental issues. However, this is a *post hoc* interpretation that should only be made with great caution.

International comparisons across 17 countries showed substantial variation in MHL, with Germany reporting the lowest levels of ability to access, understand, evaluate, and apply online health information (65). Compared with the German general population, where 76% demonstrated inadequate or problematic MHL (46), participants in our study showed comparatively high MHL (mean score of approximately 23 out of a possible 32 points). This may be explained by the fact that school teachers generally have high educational attainment and social status, both associated with higher MHL (46). Their role as multipliers and responsibility for others may also encourage active engagement with health information, much of which is accessed through digital media. In our study, MHL was inversely associated with EMF risk perception, indicating that participants with greater ability to find, access, and evaluate online health information perceived lower EMF-related risks. It needs to be highlighted, however, that there was no evidence for an association of MHL and risk perception after adjustment for TA and conspiracy belief suggesting that these two variables are more dominant correlates than MHL. In fact,an inverse association was observed between TA and EMF risk perception in both professions that remained even after adjustment for conspiracy belief and MHL. Overall, EMF risk perception among school and nursery school teachers appears to vary according to both technology affinity and, possibly to a lesser extent, digital health literacy.

In addition, also trust in (health) authorities like the WHO, the Federal Office for Radiation Protection, the Federal government or science itself were associated with risk perception towards EMF. As trust in this regard increased, the perception of EMF risk declined. These results are consistent with a study conducted in 2025 among the general populations of Germany and Greece –which examined risk perception and trust in national radiation protection authorities regarding high-frequency EMF—as well as with the results of studies conducted among physicians (25, 26). Further studies have also demonstrated an association between trust in regulatory authorities and the perception of EMF risks: In a Swiss study, trust in authorities was strongly associated with risk perception regarding 5G (29). If the public lacks knowledge regarding a specific topic, its risk perception is guided by trust in the responsible authorities (38). Besides, a high level of trust in authorities has been observed as a protective factor against the belief in conspiracy theories (66, 67).

### 4.2 Limitations

The explicit mentioning of the outcome “risk perception of electromagnetic fields” in the study invitation could have had an influence on the likelihood of participation in the study. School teachers and nursery school teachers who are particularly concerned about EMF might have been more likely to participate in the study out of self-interest. In contrast, it is also possible that school and nursery school teachers concerned about EMF participated less because they wanted to avoid EMF generating technologies and are therefore less likely to participate in an online survey. Accordingly, an over- or under-representation of EMF risk perception cannot be completely ruled out. Having said that, an earlier analysis correcting for selective non-participation using Multilevel Regression and Poststratification yielded little indication that the present study is noteworthily influenced by selection bias (42). The high comparability of the study population and the source population regarding socio-demographic characteristics also speaks against a high degree of selection bias.

It also needs to be mentioned that the primary outcome was obtained by a single item only. Even though this item was also used in earlier studies measuring EMF risk perception among physicians (26, 43), one may argue that it may not fully cover the construct of individual’s EMF-related risk perception, thus possibly suffering from low content validity. By its nature, it also lacks a measure of internal consistency, i.e., reliability. Developing and validating a multi-item scale to assess EMF risk perception may be a worthwhile undertaking for upcoming studies (42).

Furthermore, the validity of the results may have been compromised by ineligible access to the survey, particularly by bots. As countermeasures, a CAPTCHA test was introduced and data records clearly generated by bots were explicitly filtered out (e.g., by excluding questionnaires with a very short processing time of less than 5 min). Furthermore, an earlier analysis revealed no indication that the survey is notably influenced by answers generated by bots (42). Since recruitment took place partly online, the precise identification of participants as either school teachers or nursery school teachers cannot be absolutely guaranteed. Despite a targeted approach via selected professional associations and social networks, the questionnaire was, in principle, freely accessible. Furthermore, the use of a convenience sample precludes the determination of an exact response rate, as it remains unclear how many potential participants received the invitation.

It is also important to mention that due to the cross-sectional study design the observed associations do not necessarily imply a cause-and-effect relationship. Furthermore, the results should be generalized to school teachers and nursery school teachers in other countries only with great caution, as countries most likely have different training or study programs for entering the teaching profession. In fact, even within in Germany each federal state defines its own curriculum, which specifies whether and in how much detail the topic of EMF is covered in physics lessons.

### 4.3 Implications

Since school and nursery school teachers play a key role as multipliers of health-related information, it is of crucial importance for public health that these professional groups are able to convey precise scientific knowledge regarding potential health risks, including those possibly related to environmental factors like EMF. At the same time, practical implications should only be derived under the caveat that due to the design of our analysis we cannot determine if the observed associations are causal.

With this caveat in mind, our results might be interpreted as at least providing some evidence concerning some measures that may be helpful for the practice of risk communication. On the one hand, one may argue that it is important to counter conspiracy narratives, e.g., through early and easily understandable, transparent, and evidence based public education or through fact-based and logic-based inoculation messages that warn against misinformation or debunk it in advance (60, 61, 68). On the other hand, however, an information-based approach may fall short if trust in state actors and scientific institutions is low or non-existent. Strengthening this trust—for example, through participatory decision-making processes, community feedback systems, maximum transparency regarding the setting of limit values, and the independent funding of research—has been identified as a prerequisite for countering unfounded fears in other studies (60, 61). At the same time, increasing MHL could contribute to long-term resilience as investments in the population’s digital and health-related media literacy could empower citizens to distinguish reliable, accurate information from disinformation (60, 69).

## 5 Conclusion

Our findings underline that individuals’ appraisal of scientific evidence, in this case concerning EMF exposure, may be related not only to skills like media health literacy, but also to personal attitudes like technology acceptance, the tendency towards conspiracy beliefs or trust in organizations. Given the potential consequences for public and individual health, it may be regarded as very important to develop and implement strategies that—through empowerment, education, participation, and transparency—foster d trust in public and scientific institutions, while simultaneously countering conspiracy narratives.

## Data Availability

The data used in the present study is not openly available due to data protection regulation. Inquiries can be directed to the corresponding author.
