# Supplementary material for: Factors associated with risk perception among school and nursery school teachers concerning potential health effects of exposure to electromagnetic fields
Source: Front Public Health. 2026 Sep 18;14:1944031. doi: 10.3389/fpubh.2026.1944031 (PMC13630673; doi:10.3389/fpubh.2026.1944031)
Supplement: Supplementary file 1 [file Data_Sheet_1.PDF]

Supplementary Material

Supplementary file 1: Questionnaire

The original questionnaire is in German (below). The questionnaire was only translated to English for presentation in this additional file. German questions were translated by one person without back-translating and should not be used without further validation.

English Version

1. **Demographics and working environment**

What professional title applies to you?

☐ School teacher

☐ Nursery school teacher

☐ Other ____________

How old are you?

☐ < 20 years

☐ 20-29 years

☐ 30-39 years

☐ 40-49 years

☐ 50-59 years

☐ ≥ 60 years

What is your gender?

☐ Female

☐ Male

☐ Diverse

The sponsorship of the institution where you primarily work is…

☐ Public

☐ Private

☐ Other ___________

In which type of school do you teach most often? (This question is only displayed to school teachers.) (Note: The exact terms may vary depending on the federal state.)

☐ Elementary School

☐ Secondary/Middle School

☐ Secondary Modern School

☐ Grammar School

☐ Comprehensive School

☐ School type with multiple educational exits

☐ Free Waldorf School

☐ Special school

☐ Further Education Colleges

☐ Others ________

Which school subjects do you teach? (This question is only displayed to school teachers)

____

What age are the children you mainly work with (This question is only displayed to nursery school teachers.)

☐ < 3 years

☐ 3–6 years

☐ 7–10 years

☐ 11–14 years

☐ ≥15 years

Does the institution where you mainly work have any other special features that you would like to mention here (e.g., a specific educational concept such as Montessori, etc.)?

____

In which federal state do you mainly work?

☐ Baden-Württemberg

☐ Bavaria

☐ Berlin

☐ Brandenburg

☐ Bremen

☐ Hamburg

☐ Hesse

☐ Mecklenburg-Western Pomerania

☐ Lower Saxony

☐ North Rhine-Westphalia

☐ Rhineland-Palatinate

☐ Saarland

☐ Saxony

☐ Saxony-Anhalt

☐ Schleswig-Holstein

☐ Thuringia

How many inhabitants does the town have where the facility where you primarily work is located?

☐ < 5.000

☐ 5.000–10.000

☐ 10.000–20.000

☐ 20.000–100.000

☐ > 100.000

2. Risk perception

Note: The following questions regarding possible health effects of electromagnetic fields always assume compliance with the legally defined limits.

To what extent do you agree with the following statements?

"There are individuals who develop adverse health effects from electromagnetic fields below legal limits."

Disagree Agree

|__| |__| |__| |__| |__|

3. Electromagnetic fields, mobile networks and 5G

Please indicate to what extent you agree with the following statements:

"Compared to 3G and 4G, I will save time by using 5G services."

Disagree Agree

|__| |__| |__| |__| |__|

"Using 5G services will improve my performance and effectiveness at work."

Disagree Agree

|__| |__| |__| |__| |__|

"The use of 5G services will have more advantages than disadvantages."

Disagree Agree

|__| |__| |__| |__| |__|

"Compared to 3G and 4G, 5G services will bring more benefits."

Disagree Agree

|__| |__| |__| |__| |__|

“Overall, the use of 5G services will be beneficial.“

Disagree Agree

|__| |__| |__| |__| |__|

“Using 5G services would increase the range of my communication.“

Disagree Agree

|__| |__| |__| |__| |__|

“Compared to 3G and 4G, it will be easier to learn how to use 5G services.“

Disagree Agree

|__| |__| |__| |__| |__|

“Compared to 3G and 4G, 5G services will make it easier to do what I want to do.“

Disagree Agree

|__| |__| |__| |__| |__|

“Overall, I believe that 5G services are easier to use.“

Disagree Agree

|__| |__| |__| |__| |__|

"I believe I would find it easy to get to grips with using 5G services.“

Disagree Agree

|__| |__| |__| |__| |__|

"I would consider the 5G services to be flexible.“

Disagree Agree

|__| |__| |__| |__| |__|

"Using 5G services would not require much mental effort.“

Disagree Agree

|__| |__| |__| |__| |__|

4. **Health-related behavior**

In the next question, we want to know how easy or difficult you find it to search for and evaluate information on the internet.

Please indicate to what extent the following statements apply to you.

How easy/difficult is it for you…?

"…using the right words or search terms to find the desired information online?“

Very easy Very difficult

|__| |__| |__| |__| |__|

"… to find exactly the information you are looking for?“

Very easy Very difficult

|__| |__| |__| |__| |__|

"… to understand the information found?"

Very easy Very difficult

|__| |__| |__| |__| |__|

"… to assess how trustworthy the information found is?"

Very easy Very difficult

|__| |__| |__| |__| |__|

"… to assess whether commercial interests are behind the information provided?"

Very easy Very difficult

|__| |__| |__| |__| |__|

"… to visit different websites to check if they contain the same information on the topic?"

Very easy Very difficult

|__| |__| |__| |__| |__|

"… to assess whether the information applies to you?"

Very easy Very difficult

|__| |__| |__| |__| |__|

"… to use the information found to solve a health problem?"

Very easy | | | | Very difficult
|__| |__| |__| |__| |__|

5. **Questions of trust**

As a reminder: The questions posed here regarding potential health problems caused by electromagnetic fields always assume compliance with the legally established limits.

Please indicate the extent to which you agree with the following statements. (Answer options are randomized)

"The truth is that the connection between health problems and electromagnetic fields is being withheld from the public."

Disagree | | | | Agree
|__| |__| |__| |__| |__|

"People need to wake up and think about the fact that there is a connection between health problems and electromagnetic fields."

Disagree | | | | Agree
|__| |__| |__| |__| |__|

"Legitimate questions about the connection between health problems and electromagnetic fields are being suppressed by the government, the media, and the scientific community."

Disagree | | | | Agree
|__| |__| |__| |__| |__|

“Reporters, scientists, and government officials are involved in a conspiracy to cover up important information about the link between health problems and electromagnetic fields.“

Disagree				Agree
|__|	|__|	|__|	|__|	|__|

“An impartial, independent investigation into the link between health problems and electromagnetic fields would show that we have been widely lied to.“

Disagree				Agree
|__|	|__|	|__|	|__|	|__|

How trustworthy are the following organizations and groups of people when it comes to information about the health effects of electromagnetic fields?

World Health Organization (WHO)

Not trustworthy at all				Very trustworthy	I am not familiar with it
|__|	|__|	|__|	|__|	|__|	|__|

Federal Office for Radiation Protection

Not trustworthy at all				Very trustworthy	I am not familiar with it
|__|	|__|	|__|	|__|	|__|	|__|

Doctors' appeals

Not trustworthy at all				Very trustworthy	I am not familiar with it
|__|	|__|	|__|	|__|	|__|	|__|

Citizens' initiatives

Not trustworthy at all				Very trustworthy	I am not familiar with it
|__|	|__|	|__|	|__|	|__|	|__|

Institute for Applied Ecology

Not trustworthy at all				Very trustworthy	I am not familiar with it
|__|	|__|	|__|	|__|	|__|	|__|

Consumer Organizations

Not trustworthy at all				Very trustworthy	I am not familiar with it
|__|	|__|	|__|	|__|	|__|	|__|

In the next question, we are interested in your general views on politics in Germany. When you think of the following institutions or groups of people: How trust do you generally have in them?

Federal government

No trust				Very great trust
|__|	|__|	|__|	|__|	|__|

Politicians

No trust				Very great trust
|__|	|__|	|__|	|__|	|__|

Political parties

No trust				Very great trust
|__|	|__|	|__|	|__|	|__|

Courts

No trust				Very great trust
|__|	|__|	|__|	|__|	|__|

Large companies

No trust				Very great trust
|__|	|__|	|__|	|__|	|__|

Police

No trust				Very great trust
|__|	|__|	|__|	|__|	|__|

Armed forces

No trust				Very great trust
|__|	|__|	|__|	|__|	|__|

Media

No trust				Very great trust
|__|	|__|	|__|	|__|	|__|

Science

No trust				Very great trust
|__|	|__|	|__|	|__|	|__|

Most other people

No trust				Very great trust
|__|	|__|	|__|	|__|	|__|

6. Health and environment

Please indicate whether and how much you are concerned about various factors affecting your personal health.

Do you worry about your personal health because of…?

Air pollution

No worries at all				Great worries
|__|	|__|	|__|	|__|	|__|

Drinking water pollution

No worries at all				Great worries
|__|	|__|	|__|	|__|	|__|

Traffic noise

No worries at all				Great worries
|__|	|__|	|__|	|__|	|__|

Impacts of climate change

No worries at all				Great worries
|__|	|__|	|__|	|__|	|__|

Resistance to antibiotics

No worries at all				Great worries
|__|	|__|	|__|	|__|	|__|

Pesticides

No worries at all				Great worries
|__|	|__|	|__|	|__|	|__|

Original Version in German

1. **<u>Demographie und Arbeitsumfeld</u>**

Welche Berufsbezeichnung trifft auf Sie zu?

☐ Lehrer*in

☐ Erzieher*in

☐ Andere, bitte eintragen: ______________________

Wie alt sind Sie?

☐ Unter 20 Jahren

☐ 20-29 Jahre

☐ 30-39 Jahre

☐ 40-49 Jahre

☐ 50-59 Jahre

☐ 60 Jahre und älter

Welchem Geschlecht fühlen Sie sich zugehörig?

☐ Weiblich

☐ Männlich

☐ Divers

Die Trägerschaft der Einrichtung, in der Sie hauptsächlich arbeiten, ist...

☐ Öffentlich

☐ Privat

☐ Andere Trägerschaft, und zwar ____________

In welcher Schulform unterrichten Sie mehrheitlich? (Diese Frage wird nur Lehrer*innen angezeigt) (Hinweis: Die genauen Bezeichnungen können je nach Bundesland abweichen.)

☐ Grundschule

☐ Hauptschule/Mittelschule

☐ Realschule

☐ Gymnasium

☐ Integrierte Gesamtschule

☐ Schulart mit mehreren Bildungsabgängen

☐ Freie Waldorfschule

☐ Förderschule

☐ Berufliche Schule (z. B. Berufschule, FOS, BOS, Fachschule)

☐ Andere Schulform: ______________________

Welche Schulfächer unterrichten Sie? (Diese Frage wird nur Lehrer*innen angezeigt)

____

Welches Alter haben die Kinder, mit denen Sie hauptsächlich arbeiten? Mehrere Antworten können ausgewählt werden (Diese Frage wird nur Erzieher*innen angezeigt)

☐ Unter 3 Jahren

☐ 3-6 Jahre

☐ 7-10 Jahre

☐ 11-14 jahre

☐ 15 Jahre und älter

Weist die Einrichtung, in der Sie hauptsächlich arbeiten, weitere Besonderheiten auf, die Sie hier gerne nennen möchten (z. B. ein spezielles pädagogisches Konzept, wie Montessori, etc.)?

____

In welchem Bundesland arbeiten Sie hauptsächlich?

☐ Baden-Württemberg

☐ Bayern

☐ Berlin

☐ Brandenburg

☐ Bremen

☐ Hamburg

☐ Hessen

- ☐ Mecklenburg-Vorpommern
- ☐ Niedersachsen
- ☐ Nordrhein-Westfalen
- ☐ Rheinland-Pfalz
- ☐ Saarland
- ☐ Sachsen
- ☐ Sachsen-Anhalt
- ☐ Schleswig-Holstein
- ☐ Thüringen

Wie viele Einwohner*innen hat der Ort, in dem sich die Einrichtung, in der Sie hauptsächlicharbeiten, befindet?

- ☐ < 5.000
- ☐ 5.000–10.000
- ☐ 10.000–20.000
- ☐ 20.000–100.000
- ☐ > 100.000

2. Risikowahrnehmung

Hinweis: Die nachfolgenden Fragen zu möglichen Gesundheitsbeschwerden durch elektromagnetische Felder setzen immer voraus, dass die Einhaltung der gesetzlich festgelegten Grenzwerte gegeben ist.

Inwieweit stimmen Sie den folgenden Aussagen zu?

„Es gibt Personen, bei denen unter Einhaltung der gesetzlichen Grenzwerte Gesundheitsbeschwerden durch elektromagnetische Felder ausgelöst werden."

Stimme nicht zu				Stimme zu
|__|	|__|	|__|	|__|	|__|

3. Elektromagnetische Felder, Handynetze und 5G

Geben Sie bitte an, inwiefern Sie den folgenden Aussagen zustimmen:

“Im Vergleich zu 3G und 4G werde ich durch die Nutzung von 5G-Diensten Zeit sparen.“

Stimme nicht zu				Stimme zu
|__|	|__|	|__|	|__|	|__|

“Die Nutzung von 5G-Diensten wird meine Leistung und Effektivität bei der Arbeit verbessern.“

Stimme nicht zu				Stimme zu
|__|	|__|	|__|	|__|	|__|

“Die Nutzung von 5G-Diensten wird mehr Vorteile als Nachteile haben.“

Stimme nicht zu				Stimme zu
|__|	|__|	|__|	|__|	|__|

“ Im Vergleich zu 3G und 4G werden die 5G-Dienste mehr Nutzen bringen.“

Stimme nicht zu				Stimme zu
|__|	|__|	|__|	|__|	|__|

“ Insgesamt wird die Nutzung von 5G-Diensten vorteilhaft sein.“

Stimme nicht zu				Stimme zu
|__|	|__|	|__|	|__|	|__|

“Die Nutzung von 5G-Diensten würde die Reichweite meiner Kommunikation erhöhen.“

Stimme nicht zu				Stimme zu
|__|	|__|	|__|	|__|	|__|

“Im Vergleich zu 3G und 4G wird es einfacher sein, die Bedienung von 5G-Diensten zu erlernen.“

Stimme nicht zu				Stimme zu
|__|	|__|	|__|	|__|	|__|

“Im Vergleich zu 3G und 4G wird es mit 5G-Diensten einfacher sein, das zu tun, was ich tun möchte.“

Stimme nicht zu				Stimme zu
|__|	|__|	|__|	|__|	|__|

“Insgesamt glaube ich, dass 5G-Dienste einfacher zu bedienen sind.“

Stimme nicht zu				Stimme zu
|__|	|__|	|__|	|__|	|__|

“ Ich glaube, dass es mir leichtfallen würde, mich in der Nutzung von 5G-Diensten zurechtzufinden.“

Stimme nicht zu				Stimme zu
|__|	|__|	|__|	|__|	|__|

“ Ich würde die 5G-Dienste als flexibel empfinden.“

Stimme nicht zu				Stimme zu
|__|	|__|	|__|	|__|	|__|

“Die Nutzung von 5G-Diensten würde keine große geistige Anstrengung erfordern.“

Stimme nicht zu				Stimme zu
|__|	|__|	|__|	|__|	|__|

4. Gesundheitsbezogenes Verhalten

Bei der nächsten Frage interessiert uns, wie einfach oder schwer es Ihnen fällt, Informationen im Internet zu suchen und zu bewerten.

Bitte geben Sie an, inwiefern folgende Aussagen auf Sie zutreffen.

Wie einfach/schwierig ist es für Sie….

“…die richtigen Wörter oder Suchbegriffe zu verwenden, um online die gewünschten Informationen zu finden?“

Sehr einfach				Sehr schwierig
|__|	|__|	|__|	|__|	|__|

“… genau die Informationen zu finden, nach denen Sie suchen?“

Sehr einfach				Sehr schwierig
|__|	|__|	|__|	|__|	|__|

“… die gefundenen Informationen zu verstehen?“

Sehr einfach				Sehr schwierig
|__|	|__|	|__|	|__|	|__|

“… zu beurteilen, wie vertrauenswürdig die gefundenen Informationen sind?“

Sehr einfach				Sehr schwierig
|__|	|__|	|__|	|__|	|__|

“… zu beurteilen, ob hinter den angebotenen Informationen kommerzielle Interessen stehen?“

Sehr einfach				Sehr schwierig
|__|	|__|	|__|	|__|	|__|

“… unterschiedliche Internetseiten aufzusuchen, um zu prüfen, ob dort gleiche Informationen zum Thema stehen?“

Sehr einfach				Sehr schwierig
|__|	|__|	|__|	|__|	|__|

“… zu beurteilen, ob die Informationen auf Sie zutreffen?“

Sehr einfach				Sehr schwierig
|__|	|__|	|__|	|__|	|__|

"… die gefundenen Informationen zur Lösung eines Gesundheitsproblems zu nutzen?"

Sehr einfach				Sehr schwierig
|__|	|__|	|__|	|__|	|__|

5. **Fragen des Vertrauens**

Zur Erinnerung: Die hier gestellten Fragen zu möglichen Gesundheitsbeschwerden durch elektromagnetische Felder setzen immer voraus, dass die Einhaltung der gesetzlich festgelegten Grenzwerte gegeben ist.

Geben Sie bitte an, inwiefern Sie den folgenden Aussagen zustimmen. (Antwortoptionen sind randomisiert)

"Die Wahrheit ist, dass der Zusammenhang zwischen Gesundheitsbeschwerden und elektromagnetischen Feldern der Öffentlichkeit vorenthalten wird."

Stimme nicht zu				Stimme zu
|__|	|__|	|__|	|__|	|__|

"Menschen müssen aufwachen und darüber nachdenken, dass es einen Zusammenhang zwischen Gesundheitsbeschwerden und elektromagnetischen Feldern gibt."

Stimme nicht zu				Stimme zu
|__|	|__|	|__|	|__|	|__|

"Berechtigte Fragen über den Zusammenhang zwischen Gesundheitsbeschwerden und elektromagnetischen Feldern werden von der Regierung, den Medien und der Wissenschaft unterdrückt."

Stimme nicht zu				Stimme zu
|__|	|__|	|__|	|__|	|__|

“Reporter*innen, Wissenschaftler*innen und Regierungsbeamt*innen sind in eine Verschwörung verwickelt, um wichtige Informationen über den Zusammenhang zwischen Gesundheitsbeschwerden und elektromagnetischen Feldern zu vertuschen.“

Stimme nicht zu				Stimme zu
|__|	|__|	|__|	|__|	|__|

“Eine unparteiische, unabhängige Untersuchung des Zusammenhangs zwischen Gesundheitsbeschwerden und elektromagnetischen Feldern würde zeigen, dass wir in großem Umfang belogen wurden.“

Stimme nicht zu				Stimme zu
|__|	|__|	|__|	|__|	|__|

Wie vertrauenswürdig sind die folgenden Organisationen und Personengruppen, wenn es um Informationen über gesundheitliche Wirkungen elektromagnetischer Felder geht?

Weltgesundheitsorganisation (WHO)

Überhaupt nicht vertrauenswürdig				Sehr vertrauenswürdig	Kenne ich nicht
|__|	|__|	|__|	|__|	|__|	|__|

Bundesamt für Strahlenschutz

Überhaupt nicht vertrauenswürdig				Sehr vertrauenswürdig	Kenne ich nicht
|__|	|__|	|__|	|__|	|__|	|__|

Ärzteappelle (z.B. Oberammergauer Appell, Freiburger Appell)

Überhaupt nicht vertrauenswürdig				Sehr vertrauenswürdig	Kenne ich nicht
|__|	|__|	|__|	|__|	|__|	|__|

Bürgerinitiativen gegen die Errichtung von Mobilfunksendemasten

Überhaupt nicht vertrauenswürdig				Sehr vertrauenswürdig	Kenne ich nicht
|__|	|__|	|__|	|__|	|__|	|__|

Ökoinstitut

Überhaupt nicht vertrauenswürdig				Sehr vertrauenswürdig	Kenne ich nicht
|__|	|__|	|__|	|__|	|__|	|__|

Verbraucherzentrale

Überhaupt nicht vertrauenswürdig				Sehr vertrauenswürdig	Kenne ich nicht
|__|	|__|	|__|	|__|	|__|	|__|

Bei der nächsten Frage interessieren wir uns für Ihre allgemeinen Ansichten zur Politik in Deutschland. Wenn Sie an die folgenden Institutionen oder Personengruppen denken:

Wie sehr vertrauen Sie diesen im Allgemeinen?

Bundesregierung

Kein Vertrauen				Sehr großes Vertrauen
|__|	|__|	|__|	|__|	|__|

Politiker*innen

Kein Vertrauen				Sehr großes Vertrauen
|__|	|__|	|__|	|__|	|__|

Politische Parteien

Kein Vertrauen				Sehr großes Vertrauen
|__|	|__|	|__|	|__|	|__|

Gerichte

Kein Vertrauen				Sehr großes Vertrauen
|__|	|__|	|__|	|__|	|__|

Große Wirtschaftsunternehmen

Kein Vertrauen				Sehr großes Vertrauen
|__|	|__|	|__|	|__|	|__|

Polizei

Kein Vertrauen				Sehr großes Vertrauen
|__|	|__|	|__|	|__|	|__|

Bundeswehr

Kein Vertrauen				Sehr großes Vertrauen
|__|	|__|	|__|	|__|	|__|

Medien

Kein Vertrauen				Sehr großes Vertrauen
|__|	|__|	|__|	|__|	|__|

Wissenschaft

Kein Vertrauen — Sehr großes Vertrauen

|__| |__| |__| |__| |__|

Die meisten Menschen

Kein Vertrauen — Sehr großes Vertrauen

|__| |__| |__| |__| |__|

6. Gesundheit und Umwelt

Geben Sie bitte an, ob und wie stark Sie sich wegen verschiedener Einflüsse auf Ihre persönliche Gesundheit Sorgen machen.

Machen Sie sich persönlich gesundheitliche Sorgen wegen …?

Luftverschmutzung

Kein Vertrauen — Sehr großes Vertrauen

|__| |__| |__| |__| |__|

Trinkwasserbelastung

Kein Vertrauen — Sehr großes Vertrauen

|__| |__| |__| |__| |__|

1

Verkehrslärm

Kein Vertrauen				Sehr großes Vertrauen
|__|	|__|	|__|	|__|	|__|

Auswirkungen des Klimawandels

Kein Vertrauen				Sehr großes Vertrauen
|__|	|__|	|__|	|__|	|__|

Antibiotikaresistenzen

Kein Vertrauen				Sehr großes Vertrauen
|__|	|__|	|__|	|__|	|__|

Pestiziden

Kein Vertrauen				Sehr großes Vertrauen
|__|	|__|	|__|	|__|	|__|
